# Epidemiology, clinical characteristics, and treatment outcomes of leishmaniasis in Qatar: A retrospective study

**DOI:** 10.5339/qmj.2024.51

**Published:** 2024-12-26

**Authors:** Mohammed Abukhattab, Joanne Daghfal, Mohammed Adam, Samar A Hashim, Fathima Koolikkad Mohammed, Muna Al-Maslamani

**Affiliations:** 1 Hamad Medical Corporation, Doha, Qatar *Email: mabukhattab@gmail.com

**Keywords:** leishmaniasis, epidemiology, clinical characteristics, treatment outcomes

## Abstract

**Background:**

Leishmaniasis is an emerging tropical infectious disease in Qatar. It is caused by the protozoan parasite of the *Leishmania* genus, which is endemic in many regions of the world, including the Middle East. In Qatar, there is limited data about this disease, which is hindering the efficient planning and implementation of disease prevention and control measures. Therefore, to address this knowledge gap, we conducted a retrospective study to gather information on the prevalence, clinical characteristics, as well as outcomes of leishmaniasis treatment. The primary objective of this study is to provide a comprehensive analysis of leishmaniasis cases in Qatar over seven years (2016–2022). The findings of this study offer valuable insights that can guide the development of a national registry and treatment program for leishmaniasis in Qatar.

**Methods:**

Using a retrospective cross-sectional study design, clinical and epidemiological data of all documented cases of leishmaniasis in Qatar between 2016 and 2022 were collected from electronic medical records at Hamad Medical Corporation (HMC).

**Results:**

A total of sixty-eight cases of leishmaniasis were detected in Qatar between 2016 and 2022. Males were predominant (69.12%) with a median age of 33 years. Our study revealed a higher incidence of leishmaniasis among individuals of Syrian nationality, followed by Pakistani and Sudanese, thus promoting further investigation into the underlying factors contributing to this health disparity. Our findings revealed important epidemiological trends, highlighted key clinical characteristics, identified risk factors associated with susceptibility to leishmaniasis, and reported treatment outcomes.

**Conclusion:**

This retrospective study presents the first comprehensive analysis of leishmaniasis in Qatar, shedding light on the epidemiology, clinical characteristics, and treatment outcomes of the disease. The data generated from this study can serve as a guide for authorities in establishing a national registry and treatment program for leishmaniasis in Qatar. Implementing these measures will contribute to the effective management and control of leishmaniasis, ultimately improving patient outcomes and public health in the country.

## 1. Background

Leishmaniasis is a tropical disease in humans caused by vector-borne hemoflagellate parasites and transmitted by the bite of parasite-infected sand flies (*Phlebotomus* spp., vectors of Old World leishmaniasis, and *Lutzomyia* spp., vectors of the Western Hemisphere). The disease is one of the most neglected tropical diseases (NTDs) and is known as the poor man’s disease. It affects approximately 98 nations in Asia, Africa, the Middle East, Central America, and South America. More than 350 million people live within the area of transmission.^1-3^

Leishmaniases have a dynamic epidemiology, indicating that the conditions required for transmission constantly shift due to changes in the environment, population behavior, socioeconomic level, and immunogenic profile of the infected human populations. *Leishmania* spp. is known to cause several different disease manifestations, including visceral leishmaniasis (VL), cutaneous leishmaniasis (CL), mucocutaneous leishmaniasis (MCL), diffuse cutaneous leishmaniasis (DCL), and less commonly, leishmaniasis recidivans (LR).^4-7^ Globally, estimates of the annual incidence of CL vary from 0.7 to 1.2 million cases per year with 95% of the disease accounting in America, the Mediterranean basin, the Middle East, and Central Asia. There are 0.2–0.4 million cases of VL reported per year; however, their numbers have decreased significantly from previous estimates. In 2020, the World Health Organization (WHO) reported that more than 90% of cases from seven countries, including Brazil, Ethiopia, India, Kenya, Somalia, South Sudan, and Sudan, are linked to VL. In 2016, more than 84% of the cases were reported from 10 different countries, which includes Yemen, Algeria, Afghanistan, Brazil, Colombia, Iraq, Pakistan, Peru, Syria, and Tunisia.^8^

Leishmaniasis represents a major public health concern in the Eastern Mediterranean region. In Saudi Arabia, CL is the most reported type of leishmaniasis; however, because of leishmaniasis control measures, there has been a significant drop in the cases since the 1990s, from 1000 to less than 600 in 2021.^9^ In Yemen, a total of 6416 cases of CL were reported from 2019 to 2022. In the Syrian Arab Republic, 89,357 cases were reported in the year 2019.^10^

The state of Qatar is a peninsular region on the northeastern coast of the Arabian Gulf with an estimated population of 2,956,261 million as per the census of April 2023.^11^ Foreign workers make up the majority of the population, which comprises South Asians, with those from India alone estimated to be around 700,000.^12^ Egyptians and Filipinos are the largest non-South Asian migrant group in Qatar. There are no reported cases of endogenous transmission in Qatar; all reported cases are imported from endemic countries. However, this poses a risk for the country due to the substantial number of immigrant workers who represent 88% of Qatar’s total population. The incubation period of the disease can span from 10 days to 24 months, with an average duration of 2–6 months. The infected patients may not exhibit the symptoms until a significant time has passed since exposure. Thus, these patients may present to our healthcare system after developing a confirmed diagnosis. The risk of disease transmission in Qatar could be exacerbated by factors like the substantial increase in immigration, notably among migrant workers. Even with the increased susceptibility, there is limited data about the disease in Qatar. To address this gap, epidemiological studies are crucial for planning effective strategies to control the spread of this disease within the country. Thus, our study aims to conduct a comprehensive analysis of all cases of leishmaniasis diagnosed in the country since 2016. By understanding the epidemiological patterns, clinical signs and symptoms, risk factors, and treatment options, this study will help in providing evidence-based policies for control and management of the disease, thus reducing its burden. The recognized risk factors for leishmaniasis include migration, poverty, malnourishment, and inadequate hygiene. Furthermore, this study, being the first of its kind in the region, will improve our understanding of leishmaniasis in the state and aid in establishing targeted preventive measures to prevent future outbreaks.

## 2. Methods

A retrospective review of electronic medical records was undertaken to identify all documented cases of leishmaniasis in Qatar between 2016 and 2022. Hamad Medical Corporation (HMC) is the main healthcare provider in the country with 15 specialty hospitals. It caters to the population of Qatar and epidemiologically represents both locals and expatriates in the country. The included study population comprised all cases diagnosed with leishmaniasis during the study period in all HMC hospitals. The study relied on results from our in-house laboratory, which is the main reputable healthcare provider in Qatar, and this information is appropriate for this type of research. The variables of interest in our study included age, sex, nationality, date of diagnosis, type of leishmaniasis, history of immunosuppressive disease or immunosuppressive treatment, as well as history of contact with an infected animal suspected of infection and travel to an endemic country according to WHO’s current guidelines. Data regarding patient demographics, clinical features, treatment modalities, and outcomes were collected and analyzed.

Quantitative variables were expressed as the median and interquartile range (IQR) used to describe the central tendency and spread of continuous variables, such as patient age, while categorical variables were presented as absolute values and proportions. Frequencies and percentages are used to describe the distribution of leishmaniasis cases by nationality, gender, clinical presentation, treatment modalities, and other categorical variables. All analyses were performed with Stata Statistical Software, version 16.1 (StataCorp., College Station, TX, USA).

It’s important to note that the study does not involve hypothesis testing or inferential statistics, as it is primarily focused on describing the characteristics and patterns of leishmaniasis cases in Qatar. Instead, it provides a snapshot of the epidemiology and clinical features of the disease in the region over a specific time frame.

## 3. Results

A total of 68 cases of leishmaniasis were diagnosed in Qatar over 6 years between 2016 and 2022; 60 (88.23%) cases were CL, 7 (10.29%) cases were VL, and 1 (1.47%) was MCL.

Males were more prone to leishmaniasis accounting for 69.12% (47 cases) of the cases, and 30.88% (21 cases) were females. The male to female ratio was 2.2:1. The median age of the cases was 33 (10–45 years).

**Figure 1.**
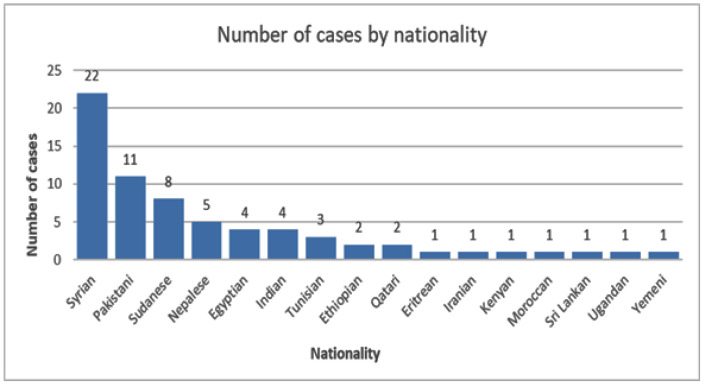
Distribution of cases according to nationality.

Among the cases, the majority were non-Qataris, and only 2 cases were Qataris. Syrians constituted the largest group accounting for 22 cases (32.35%), followed by Pakistanis with 11 cases (16.18%) and Sudanese with 8 cases (11.76%). Nepalese accounted for 5 cases (7.35%), while Egyptians and Indians had 4 cases each (5.88%) and Ethiopians accounted for 2 cases. Additionally, there was 1 case each from Eritrea, Iran, Kenya, Morocco, Sri Lanka, Uganda, and Yemen, each representing 1.47% of the total number of cases (Figure 1).

In our study, 12 patients (17.65%) had other comorbidities, while3 patients (4.41%) were found to be immunosuppressed.

Furthermore, 47 patients (69.12%) reported a history of travel to the endemic areas, and 2 (2.94%) reported exposure or contact with animals (Table 1).

**Figure 2.**
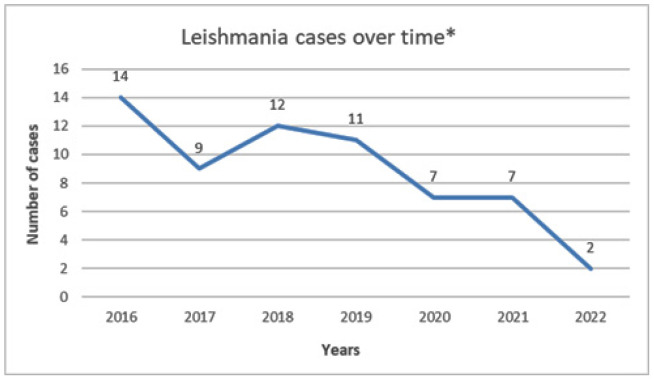
*Leishmania* case per year. *6 Cases with missing medical records.

The data reveal a gradual reduction in the number of diagnosed cases over the study period. Precisely there were 14 cases diagnosed in 2016 (20.59%), followed by 9 cases in 2017 (13.24%), 12 cases in 2018 (17.65%), and 11 cases in 2019 (16.18%). Consequently, there were 7 cases each in 2020 and 2021 (10.29%) and 2 cases in 2022; moreover, the medical records were missing for 6 cases (Figure 2).

The most prevalent presentation amongst patients with leishmaniasis was skin lesions observed in 54 cases (79.41%), followed by fever in 8 cases (11.76%), while GI symptoms, loss of appetite, and fatigue were reported in 7 cases (10.29%) (Table 2).

Nine patients (13.23%) received intravenous Amphotericin B, and one patient (1.47%) underwent oral systemic therapy. Local therapy was utilized for 45 patients (66.18%), while two patients (2.94%) were treated with intra-lesional Pentostam. However, treatment mode documentation was missing for 11 cases (14.71%) (Table 3).

Among the cases studied, 15 patients (22.06%) were successfully cured without any sequalae, while7 patients (10.29%) were cured but experienced sequalae. Also, two patients reported significant improvement, and two patients are currently under follow-up. For the remaining cases, follow-up data was either lost or missing (Table 4).

Among the seven patients diagnosed with VL, the majority were of Nepalese origin (4 patients) and one patient each from Ethiopia, Kenya, and Sudan.

Two of these patients presented with other co-morbidities, and none of them were immunosuppressed. All patients originated from endemic countries with no documented animal exposure. The most common clinical manifestations of VL patients included fatigue and weight loss followed by fever and loss of appetite.

Laboratory findings indicated a median white blood cell (WBC) count of 1.5 × 10^3^/µL, hemoglobin levels of 9.7 g/dL, and platelet count of 56 × 10^3^/µL. Other laboratory parameters, including liver function test and coagulation profiles, were in the normal range. Six VL patients were treated with IV Amphotericin B and four patients were cured without any sequelae. The details of treatment outcomes of VL subgroup patients are shown in Table 5.

## 4. Discussion

In this research article, we aimed to conduct a comprehensive review of all documented cases of leishmaniasis in Qatar since 2016 and provide insights into the epidemiology, clinical features, risk factors, and treatment outcomes of the disease. The data for the study were obtained through a retrospective review of electronic medical records from HMC, which is the main health service provider in the country. The insights gained from this study will help in the establishment of preventive measures and potentially contribute to the development of a national registry for leishmaniasis in Qatar.

Leishmaniasis has a long history, dating to 2,500 B.C., with ancient writings containing several primitive descriptions of the disease and molecular findings uncovering evidence from ancient archeological materials.^13^

Leishmaniasis is a group of vector-borne diseases caused by more than 20 species of protozoan parasites from the *Leishmania* genus. These parasites are transmitted between humans and other mammalian hosts through the bites of phlebotomine sandflies.^14^ At least 23 species of *Leishmania* have been associated with humans.^15^ Humans are usually infected incidentally when they enter endemic areas.

Leishmaniasis is considered a neglected tropical disease and is listed among the NTDs by the WHO and the Centers for Disease Control and Prevention (CDC). It is estimated to cause a significant disease burden, ranking ninth among individual infectious diseases.

The exact number of leishmaniasis cases is unknown, but it is estimated that annually there are 0.2–0.4 million cases of VL and 0.7–1.2 million cases of CL. CL is the most common form of the disease, with approximately 2 million cases occurring each year.^1^

Leishmaniasis occurs in 98 countries, primarily located in Southern Europe, Africa, Asia, South Asia, and South and Central America; however, it is not found in Australia or the Pacific Islands, reported mainly in rural areas than in urban areas.^1-3^

Geographically, leishmaniasis is classified into two main regions: the Old World (the Eastern Hemisphere) and the New World (the Western Hemisphere). In the New World, the disease is prevalent in certain areas of Mexico, Central America, and South America. Additionally, sporadic cases of CL have been reported in the United States specifically in the region of Texas and Oklahoma.^16^

The *Leishmania* species are known to cause various disease manifestations, including VL, CL, MCL, DCL, and less commonly, LR. Importantly, leishmaniasis is not directly transmitted from person to person; instead, it is primarily spread through the bites of infected sandflies.^4-7^

*Leishmania* species exhibit many similarities in terms of genetics, mode of transmission, biochemistry, molecular biology, immunobiology, and susceptibility to drugs. Notably, the most significant divergence occurs between the cutaneous and visceralizing species of *Leishmania*.

The subgenus *Leishmania* is distributed across both the Old and the New World, whereas the subgenus *Viannia* is exclusively found in the New World. In the Western Hemisphere, multiple species commonly infect humans, including *Leishmania (Leishmania) amazonensis, Leishmania (Viannia) braziliensis, L. (V.) peruviana, L. (V.) colombiensis, L. (L.) donovani, L. (L.) garnhami, L. (V.) guyanensis, L. (L.) infantum chagasi, L. (V.) lainsoni, L. (V.) lindenbergi, L. (L.) mexicana, L. (V.) naiffi, L. (V.) panamensis, L. (L.) pifanoi, L. (V.) shawi, and L. (L.) venezuelensis*. In contrast, the Eastern Hemisphere exhibits a smaller diversity of species that infect humans, including *L. (L.) donovani, L. (L.) infantum, L. (L.) aethiopica, L. (L.) major, and L. (L.) tropica*.^17^

Cutaneous leishmaniasis (CL) is the most common leishmanial syndrome worldwide and is frequently encountered among patients in North America.^18^ It is also considered an endemic disease in the Middle East and North Africa (MENA) region, with countries like Syria reporting a high incidence rate. Despite efforts to establish national control programs for vector containment and infection treatment, the disease continues to spread.

In VL, the *Leishmania* parasites have the ability to spread and disseminate throughout the body’s reticuloendothelial system. If left untreated, VL can be potentially life-threatening. It is crucial to diagnose and treat VL promptly to prevent complications and improve patient outcomes.

It is important to note that there are currently no vaccines or chemoprophylaxis available for the prevention of leishmaniasis. However, using personal protective measures such as repellents and mosquito nets can significantly minimize exposure to sand fly bites, which is the primary mode of leishmaniasis transmission. These preventive measures are particularly important in endemic areas.

Leishmania, the parasite that causes leishmaniasis, has been reported in 12 countries within the MENA region, encompassing a total of 20 countries.^19^ The MENA region faces many challenges like the ongoing conflicts, limited resources in veterinary and public health sectors, lack of adequate control programs, uncontrolled animal transportation across “open” borders, and poverty, all of which contribute to the spread of leishmaniasis and other zoonotic diseases.^20^

Our study identified 68 patients diagnosed with leishmaniasis over a period of 6 years. This finding represents a significant incidence rate in an area like Qatar where local transmission is uncommon. This observation may be related to the demographic composition of the country characterized by a predominant population of non-expatriates. Most of the positive leishmaniasis cases in our study were from Syria followed by Pakistan and Sudan. This aligns with existing literature from Syria which has shown a significant increase in leishmaniasis cases from 17,709 in 2007 to 82,275 in 2018.^21^ Furthermore, a study conducted in Europe showed that leishmaniasis was the most reported infectious disease among Syrian migrants.^21^ In a study conducted in a small region of Khyber Pakhtunkhwa (KPK) in Pakistan, 439 people tested positive for the disease.^22^ Therefore, this proves that it is important to follow strict surveillance for migrant workers coming from endemic countries. Effective monitoring and reporting of diseases in the migrant population will aid in preventing local transmission, especially considering that they comprise 88% of Qatar’s population. Understanding the epidemiology and transmission dynamics is essential for developing public health strategies to mitigate the impact of leishmaniasis among migrant populations.

This number merely represents the surface of the issue, highlighting that the incidence can continue to persist in high numbers within the endemic area.

In our study, the majority of the patients presented with skin lesions were diagnosed with CL based on epidemiological and clinical manifestations. Out of the total number of patients, 60 cases (88.2%) patients received follow-up and treatment at the dermatology clinic, where local measures were used. This included topical treatments using antibiotics, antifungals, and steroids in 34 cases (50%), as well as cryotherapy and liquid nitrogen application in 10 cases (14.71%). Additionally, intra-lesional Pentostam and liquid nitrogen were used in 2 cases (2.94%), respectively. Moreover, systemic therapy (IV Amphotericin B) treatment was done for the 6 cases diagnosed as VL.

Male sex represents the majority of reported cases in Qatar (69%); this can be related to socio-cultural or biological factors. This is also consistent with reports from Iraq, Yemen, and Pakistan, where males had a higher incidence of the disease compared to females.^8-10^ Most cases recall a history of travel to an endemic area (69%), but the rest might have failed to recall remote travel. A study from Saudi Arabia pointed out several factors that led to the emergence of CL in the country, including rapid urbanization, climate change, and human migration.^9^ In another study conducted in Austria, 83 of the 146 cases of CL had a travel history before the development of the disease.^23^ Thus, the factors contributing to the increasing number of leishmaniase cases in both endemic and non-endemic areas are an increase in population migration, travel activities of humans and animal hosts, and growth, as well as the spread of sandflies due to favorable climatic conditions.

Medical comorbid conditions or immunosuppression (4%) may not directly contribute as a risk factor for developing *Leishmania* infection. However, it could potentially influence the clinical outcome. To address this, a longitudinal retrospective review with a large sample size is necessary. Unfortunately, our study was unable to address this issue due to the high proportion of missing data on follow-up.

CL treatment primarily involved local measures, whereas IV Amphotericin B served as the first-line treatment option for VL. Our study revealed a significant number of patients (48.53%) who lost follow-up which indicates the absence of a centralized registry for this disease. This is possibly influenced by demographic changes in a rapidly growing country. This is especially evident in the last few years leading up to the preparation for FIFA World Cup 2022, which witnessed a significant increase in the workforce from around the globe.

The most prevalent clinical presentations observed among VL cases were fever, fatigue, loss of appetite, and weight loss. This is consistent with other studies on VL which reported similar symptoms. Laboratory findings revealed thrombocytopenia hepatomegaly and splenomegaly, aligning with findings reported in similar studies.^24^ In other studies, patients had elevated liver enzymes, anemia, and abdominal discomfort; however, in our study, the laboratory parameters, including liver function tests and coagulation profiles, were in the normal range.^25^

Treatment of VL involves a combination of systemic therapy and local measures. The Food and Drug Administration (FDA)-approved medication is intravenous liposomal amphotericin B (L AmB) which is effective in combating parasites and controlling the infection. It is essential to administer the appropriate dose and duration of treatment as prescribed by healthcare professionals.^26^

The recommended treatment options for CL vary based on the geographical region and the species of *Leishmania* responsible for the infection.

In general, the first-line treatment for CL is oral miltefosine, which is an antiparasitic medication. Miltefosine has been approved by the FDA for the treatment of CL, MCL, and VL caused by certain species. It is considered an effective and convenient treatment option for CL.

Alternatively, depending on the severity and location of the lesions, other treatment options include topical therapies such as topical paromomycin, thermal therapy (using heat or lasers to destroy the lesions), or cryotherapy (freezing the lesions with liquid nitrogen).^27-29^

In our study, 64% of patients were treated with local measures in the dermatology clinic. This included the use of topical antibiotics, antifungal medications, steroids, cryotherapy, and liquid nitrogen application. The intra-lesional Pentostam and liquid nitrogen were used in a small percentage of cases.

There were no reported mortality cases among all the patients who completed their follow-up, including those with VL. This is significantly lower than the 11–12% mortality rates reported in other studies.^30^

### 4.1. Study limitations and potential sources of bias

This study is subject to some limitations.

This study relies on historical medical records which can introduce recall bias and may lead to incomplete or inaccurate data. Patients’ records may not have been consistently documented over time. Missing data in follow-up information can limit the assessment of treatment outcomes and the long-term impact of leishmaniasis on patients. This may introduce bias and affect the study’s ability to draw comprehensive conclusions.

The study lacks detailed demographic and clinical information of the recruited patients in the study, including severity of cases, specific treatment regimens, and the nature of sequelae in cured patients.

Furthermore, there is a lack of comprehensive demographic information, such as socioeconomic status, educational background, or occupation, which could be relevant in understanding risk factors and disease patterns.

Due to the unique demographics of Qatar, with a significant proportion of the population consisting of expatriates from endemic regions, the results of the study may not be generalized.

## 5. Conclusion

The combination of factors such as conflicts, poverty, lack of veterinary and public health infrastructure, and the population demographic in endemic areas contributes to the ongoing spread of leishmaniasis. Efforts to control and manage the disease, including vector control, early diagnosis, and appropriate treatment, are crucial in mitigating the impact of leishmaniasis in these regions.

The results of the study highlight the presence of leishmaniasis in Qatar, providing baseline data that can contribute to future research in the country. This data will be valuable in determining the need for a national *Leishmania* eradication and control program, focusing on enhancing treatment and follow-up procedures for individuals diagnosed with the disease. It is important to note that most cases in Qatar are CL and imported from endemic countries, with no local transmission reported. Our recommendation includes launch of public health awareness campaigns targeting both residents and expatriates in Qatar. Those campaigns should focus on educating the population about the risks of leishmaniasis, preventive measures, and the importance of early reporting of symptoms.

In our study, no mortality cases were reported among the patients who completed their follow-up, including those with VL. This contrasts with mortality rates reported in other studies which ranged from 11% to 12%. The most common presentation among VL cases in our study was fever, fatigue, loss of appetite, and weight loss, which is consistent with findings from previous studies. Additionally, laboratory findings showed thrombocytopenia and hepatosplenomegaly, which are also consistent with other studies as well.

The decrease in leishmaniasis incidence from 14 to 2 cases per year could be attributed to several factors. Ongoing control measures in endemic countries may have contributed to this decline as well as potential changes in the population demographics in Qatar in the recent years. Additionally, the implementation of travel restrictions during the COVID-19 pandemic may have played a role in reducing the spread of the disease.

However, healthcare professionals in Qatar should be aware of this neglected disease and be able to recognize, treat, and prevent complications using advanced diagnostic technology.

In conclusion, this study highlights the epidemiological landscape of leishmaniasis prevalence in Qatar. To address the challenges and improve the management of leishmaniasis in Qatar, it is crucial to establish a national registry system that can effectively collect and track data on leishmaniasis cases. This would allow for enhanced surveillance, accurate reporting of cases, and close monitoring of treatment outcomes. With a comprehensive registry system in place, healthcare authorities can establish targeted treatment programs and interventions to address the burden of leishmaniasis in the country. By focusing on large-scale epidemiological studies, we can understand the disease prevalence and investigate the impact of climate change on the behavior and distribution of sandfly vectors. Future research endeavors should focus on the development of targeted strategies to mitigate the local transmission of leishmaniasis in Qatar. Additionally, it is important to study the patterns of spread, influence of migration, and travel to prevent the spread of leishmaniasis to the country from endemic regions.

## Conflict of Interest Statement

The authors declare that there is no conflict of interest.

## Ethical Considerations

The study obtained ethical approval from HMC medical research committee. Informed consent was not needed because of the retrospective nature of the study, which included accessed patient record only. Patient privacy and data confidentiality were assured.

## Figures and Tables

**Table 1.** Baseline demographic and clinical characteristics.

**Variable**	***N* (%) or Median (IQR)**
Age	33.00 (10.00–45.00)
**Sex**	
Female	21 (30.88%)
Male	47 (69.12%)
Comorbidities	12 (17.65%)
Immunosuppression	3 (4.41%)
Travel to endemic area	47 (69.12%)
Animal exposure	2 (2.94%)
**Type of infection**
Cutaneous leishmaniasis	60 (88.24%)
Visceral leishmaniasis	7 (10.29%)
Mucocutaneous leishmaniasis	1 (1.47%)

Data are presented as median (IQR) for continuous measures, and *N* (%) for categorical measures.

**Table 2.** Clinical presentation.

**Symptoms**	***N* (%)**
Fever	8 (11.76%)
Fatigue	7 (10.29%)
Loss of appetite	7 (10.29%)
Weight loss	6 (8.82%)
GI symptoms	7 (10.29%)
Skin lesions	54 (79.41%)

Data are presented as *N* (%).

**Table 3.** Treatment type.

**Treatment**	***N* (%)**
Local therapy	45 (66.18%)
Missing	11 (14.71%)
Amphotericin B IV	9 (13.23%)
Intra-lesional Pentostam	2 (2.94%)
Oral systemic therapy	1 (1.47%)

Data are presented as *N* (%).

**Table 4.** Treatment outcome.

**Outcome**	***N* (%)**
Loss to follow up	33 (48.53%)
Cured without sequelae	15 (22.06%)
Unknown	9 (13.24%)
Cured with sequelae	7 (10.29%)
Improved	2 (2.94%)
Undergoing follow-up	2 (2.94%)

Data are presented as *N* (%).

**Table 5.** VL subgroup demographic, clinical characteristics, and treatment outcome.

	***N* (%) or Median (IQR)**
Age	36.00 (25.00–45.00)
**Sex**
Male	7 (100.00%)
**Nationality**
Nepalese	4 (57.14%)
Ethiopian	1 (14.29%)
Kenyan	1 (14.29%)
Sudanese	1 (14.29%)
Presence of Comorbidities	2 (28.57%)
No HX of immunosuppression	7 (100.00%)
Travel to endemic area	7 (100.00%)
No animal exposure	7 (100.00%)
Fever	5 (71.43%)
Fatigue	6 (85.71%)
Loss of appetite	5 (71.43%)
Weight loss	6 (85.71%)
GI symptoms (abdominal pain or distension or *N* or	5 (71.43%)
Skin lesions	2 (28.57%)
WBC	1.50 (0.90–2.40)
Hb	9.70 (8.50–11.20)
Platelets	56.00 (14.00–72.00)
Creatinine	66.00 (61.00–82.00)
BUN	4.00 (2.60–4.90)
ALT	25.00 (15.00–43.00)
AST	38.00 (36.00–43.00)
ALK. PHOS.	143.00 (91.00–711.00)
INR	1.15 (1.10–1.50)
**US abdomen**
Hepatosplenomegaly	4 (57.14%)
Ascites	1 (14.29%)
Not done	1 (14.29%)
Missing	1 (14.29%)
**Treatment**
Parenteral systemic therapy	6 (85.71%)
Missing	1 (14.29%)
**Outcome**
Cured without sequelae	4 (57.14%)
Loss to follow up	2 (28.58%)
Cured with sequelae	1 (14.29%)

Data are presented as median (IQR) for continuous measures, and *N* (%) for categorical measures.
